# The Role of Different Methods in Defining Cardiometabolic Risk and Metabolic Syndrome in Women with Polycystic Ovary Syndrome

**DOI:** 10.3390/life13101959

**Published:** 2023-09-25

**Authors:** Nihan Çakır Biçer, Asime Aleyna Ermiş, Dilşat Baş

**Affiliations:** 1 Department of Nutrition and Dietetics, Faculty of Health Sciences, Acıbadem Mehmet Ali Aydınlar University, Icerenkoy Mah., Kayisdagi Cad. No. 32, 34752 Atasehir, Istanbul, Türkiye; aleyna.ermis@acibadem.edu.tr; 2 Department of Nutrition and Dietetics, Faculty of Health Sciences, İstanbul Galata University, Evliya Çelebi Mah., Meşrutiyet Cad. No. 62, Tepebaşı, 34425 Beyoğlu, Istanbul, Türkiye; dytdilsatbas@gmail.com

**Keywords:** polycystic ovary syndrome, metabolic syndrome, visceral adiposity, body mass index, a body shape index, body roundness index, dysfunctional adiposity index, lipid accumulation index, visceral adiposity index

## Abstract

Polycystic ovary syndrome (PCOS) is one of the most frequent endocrine illnesses, often accompanied by visceral adiposity and metabolic syndrome (MetS). Visceral adiposity is an accurate predictor of MetS and cardiometabolic risk. This study aims to evaluate different anthropometric indices that can be used in PCOS and MetS risk assessment. A total of 66 women with PCOS (50%) and 66 controls (50%) were included, and clinical and biochemical parameters were evaluated. The body mass index (BMI), body shape index (ABSI), body roundness index (BRI), dysfunctional adiposity index (DAI), lipid accumulation (LAP) index, and visceral adiposity index (VAI) were calculated. The means of all indices were higher in the PCOS group (*p* < 0.05). The marker with the lowest discriminatory ability for PCOS and MetS was ABSI (AUC = 0.762 and AUC = 0.714, respectively, *p* = 0.000). According to the multivariate logistic regression model, the VAI and WC are strong predictors of PCOS (AUC, 98%; accuracy, 92%; sensitivity, 92%; and specificity, 91%), and WC, LAP index, and BRI are strong predictors of MetS (AUC, 0.95%; accuracy, 86%; sensitivity, 83%; and specificity, 88%). The use of different anthropometric indices in the detection of PCOS and MetS may allow for early diagnosis and treatment, and are simple and cost-effective.

## 1. Introduction

Polycystic ovary syndrome (PCOS) is one of the most frequent endocrine illnesses, with a global incidence of 6–20%, and relates to hyperandrogenemia and anovulation in women, as well as long-term health [1,2,3]. PCOS, an endocrinological syndrome, is diverse, common, and associated with changes in reproductive function. Ovulatory dysfunction, a critical component in the etiology of PCOS, reduces fertility in women with PCOS. However, regardless of their ovulatory status, people with PCOS exhibit changes in several reproductive processes [4]. The vital biofluid identified as follicular fluid plays a role in reproductive processes and creates the ideal environment for oocyte development. It includes metabolites that are produced during the process and are essential for the development of the oocyte. Alterations in endometrial competence as well as oocyte and embryo quality have been noted in PCOS patients [5]. Beyond changes affecting multiple reproductive mechanisms, PCOS has additionally been demonstrated to be a risk factor for higher pregnancy problem rates, including preterm birth, perinatal fetal mortality, pre-eclampsia, gestational diabetes, and gestational hypertension [6].

Although PCOS diagnostic and treatment guidelines have been recently published, these guidelines are somewhat limited in scope, which may result in inconsistent guidance for clinicians and patients in clinical practice [7,8]. PCOS is often accompanied by metabolic syndrome (MetS), and both diseases increase the incidence of central and visceral adiposity, obesity, abnormal glucose metabolism, diabetes mellitus (DM), abnormal lipid levels, increased blood pressure, and cardiovascular disease [9,10,11]. MetS affects up to half of all PCOS women, and the prevalence varies depending on region and ethnicity [12]. The existence of MetS and abdominal obesity in PCOS have been proven to be causally related [13].

Visceral obesity has been related to proinflammatory activity, DM, dyslipidemia, hypertension, atherosclerosis, and mortality [14]. According to clinical and epidemiological data, approximately half of women with PCOS have body mass index (BMI) values above normal [15]. Furthermore, visceral obesity affects 40–85% of women with PCOS, and MetS components (insulin resistance, dyslipidemia, and hypertension) are additionally observed in this group [16]. Visceral obesity develops in both normal and overweight women with PCOS [9]. The BMI is inadequate for risk factor assessment, as it does not directly measure body fat percentage and makes a poor distinction between total body fat, and lean or bone mass [17]. According to anthropometric studies that include waist circumference (WC) measurements, women with PCOS have a distribution of abdominal fat independent of their BMI [18]. Visceral obesity is a top-notch predictor of MetS and cardiovascular (CV) risk [19]. In the assessment of visceral obesity, WC includes both subcutaneous and visceral fat tissues, resulting in inaccuracy in distinguishing visceral and subcutaneous adipose tissue in the abdomen. Visceral adipose tissue (VAT) is linked to metabolic disorders more closely than subcutaneous adipose tissue [20]. The International Diabetes Federation (IDF) recommends several methods for assessing VAT, including computed tomography (CT), magnetic resonance imaging (MRI), and dual-energy X-ray absorptiometry (DXA). However, due to their cost effectiveness and radiation risks, these methods are not used in clinical practice [21]. Therefore, specific anthropometric indices that also provide information about body fat percentage and visceral adiposity, such as a body shape index (ABSI) [22], the body roundness index (BRI) [23], the dysfunctional adiposity index (DAI) [24], the lipid accumulation (LAP) index [25], and the visceral adiposity index (VAI) [26], have been developed. Detailed information about the formulas of these indices and examples from the literature results in which these indices are examined are presented in Table 1.

Considering the characteristic importance of excessive body fat accumulation, it has been demonstrated that anthropometric measurements and adiposity parameters can be used in the risk assessment of PCOS and MetS. This study aimed to compare significant methods, including WC, ABSI, BRI, DAI, LAP index, and VAI, in the definition of MetS in patients with PCOS.

## 2. Materials and Methods

### 2.1. Study Design and Participants

This cross-sectional study included 132 women between the ages of 18 and 65 who applied to a private hospital in Turkey between 2017 and 2018. Participants were separated into two groups: the PCOS (+) group (*n* = 66) and the control group (*n* = 66). Patients with PCOS were diagnosed by a gynecologist using the Rotterdam 2003 criteria [43]. The control group consisted of women who were examined by a gynecologist for PCOS but were not diagnosed with PCOS. Women in both groups were referred to the nutrition and diet clinic for nutritional counseling. Digital records of all participants were reviewed retrospectively from the digital hospital information management system (Cerebral 2.0), and biochemical, clinical, and anthropometric measurements taken on the same day were evaluated. The following were not included in either group: individuals who were pregnant, lactating, under 19 years old, and over 65 years old; patients with chronic diseases such as cancer, type 1 DM, severe hypertriglyceridemia, hyperprolactinemia (prolactin, PRL ≥ 1.086 nM), hyperthyroidism (thyroid stimulating hormone, TSH ≤ 0.1 mIU/mL and/or FT4 ≥ 23 pM), overt hypothyroidism (TSH ≥ 10 µmIU/mL, free thyroxine levels, FT4 ≤ 9.0 pM); women who had used drugs that affect insulin and lipid metabolism or sex steroids in the last six months; and women with missing clinical and biochemical parameters used for research in the hospital information management system.

MetS is defined by the IDF as central obesity combined with any two of the following conditions: elevated fasting plasma glucose (>100 mg/dL) or previously diagnosed type 2 diabetes; elevated triglycerides (>150 mg/dL) or specific treatment for this lipid abnormality; and reduced HDL-C (50 mg/dL in women) or specific treatment for this lipid abnormality [44].

The study was approved by the Acıbadem Mehmet Ali Aydınlar University and Acıbadem Healthcare Institutions Medical Research Ethics Committee (ATADEK) (ATADEK 2020-23/36, 5 November 2020) and was conducted in accordance with the Declaration of Helsinki, and written informed consent was obtained from all participants.

### 2.2. Demographic Characteristics, Anthropometric Measurements, and the Calculation of Indices

Participants were asked about their chronic disease status, smoking and alcohol use, and regular physical activity. Body weight (kg), lean body mass (kg), body fat mass (kg), and body fat percentage were determined with bioelectrical impedance analysis (TANITA SC 330 ST) after 12 h of fasting. Height (cm) and WC (cm) were measured by experienced researchers using standard protocols and techniques, and the BMI was calculated [45]. The anthropometric indices ABSI [22], BRI [23], DAI [24], LAP index [25], and VAI [26] were calculated based on the formulas in Table 1.

### 2.3. Clinical and Biochemical Parameters

After at least 12 h of fasting, blood samples were collected. Blood samples including fasting plasma glucose (FPG), hemoglobin A1c (HbA1C), homeostatic model assessment for insulin resistance (HOMA-IR), insulin, triglycerides (TGs), total cholesterol (TC), HDL-C, low-density lipoprotein cholesterol (LDL-C), very-low-density lipoprotein cholesterol (VLDL-C), luteinizing hormone (LH), follicle-stimulating hormone (FSH), sex hormone-binding globulin (SHBG), testosterone, dehydroepiandrosterone sulfate (DHEAS), LH:FSH, and C-reactive protein (CRP) were analyzed. Systolic blood pressure (SBP) and diastolic blood pressure (DBP) were measured three times at 2 min intervals after 10 min of rest using a standardized automatic electronic sphygmomanometer, following American Heart Association recommendations, and the average of the measurements was taken [46].

### 2.4. Statistical Analysis

All analyses were carried out using IBM SPSS Statistics Version 23.0 software (IBM Corp, Arming, NY, USA). The sample size was calculated using the G*Power 3.1.9.4 program according to Vassilatou et al. [47]. The sample size estimate for the 2-sample *t*-test (*p* < 0.05; power of 0.95) was 120 women in total (66 women per group).

The Kolmogorov–Smirnov/Shapiro–Wilk test was used for the distribution of the variables, and age, total cholesterol, HDL-C, LDL-C, BMI, body fat percentage, and ABSI showed normal distributions. Other variables (FPG, insulin, HOMA-IR, HbA1c, FSH, LH, SHBG, testosterone, DHEAS, LHFSH, CRP, VLDL-C, TGs, SBP, DBP, WC, body fat mass, lean body mass, BRI, DAI, LAP, and VAI) did not follow normal distributions. Means and standard deviations were used to present descriptive analyses, and numbers (n) and percentages (%) were used to present categorical variables. The independent-samples *t*-test was used for normally distributed variables; the Mann–Whitney U test, for non-normally distributed variables; and the chi-square test, for categorical variables. To assess the associations among anthropometric measures, indices, and clinical and biochemical data, the Pearson test for normally distributed variables and Spearman’s test for non-normally distributed variables were utilized. ROC (Receiver Operating Characteristics) curve analysis was used to assess the ability of indices to predict the existence of PCOS and MetS. Univariate logistic regression analysis was used in the distribution of the effects (adjusted effects) of those who varied with the presence of PCOS by age and smoking/alcohol use. Multivariate logistic regression modeling was used to measure the effect of independent variables on dependent variables (PCOS and MetS). AUC values and diagnostic performance of the models created using ROC analysis were calculated. When calculating the area under the curve, a 5% type-I error level was employed to accept a statistically significant predictive value for the test variables. A *p*-value of less than 0.05 was considered statistically significant.

## 3. Results

### 3.1. Demographic Characteristics of Participants

A total of 132 participants, 66 of whom were diagnosed with PCOS (50%) and 66 who were healthy controls (50%), were included. The PCOS group (30.9 ± 7.5 years) had a lower mean age than the control group (36.0 ± 7.2 years) (*p* = 0.000). In the PCOS group, the presence of chronic disease (68% vs. 35%, *p* < 0.001) and smoking/alcohol use (56% vs. 11%, *p* < 0.001) was statistically significantly higher. Table 2 represents the demographic characteristics.

### 3.2. Anthropometric Measurements and Indices

The means of all anthropometric measurements and indices were higher in the PCOS group (*p* < 0.05 for all). The significant results did not change after adjusting for age and smoking/alcohol use (Table 3).

### 3.3. Biochemical Parameters and Blood Pressure Measurements

Mean FPG, insulin, HbA1c, HOMA-IR, FSH, testosterone, DHEAS, CRP, total cholesterol, LDL-C, VLDL-C, TGs in serum, SBP, and DBP levels were statistically higher in the PCOS group (*p* ≤ 0.05). In addition, the mean SHBG and HDL-C levels were found to be elevated in the control group (*p* < 0.05). After adjusting for age and smoking/alcohol use, the differences in FPG and FSH levels were found to be statistically insignificant (Table 4). 

### 3.4. Correlations among Biochemical, Clinical, and Anthropometric Parameters

Table 5 shows the correlations among biochemical, clinical, and anthropometric parameters. There were moderately positive correlations between the BMI, and FPG, insulin, HbA1c, HOMA-IR, DHEAS, CRP, total cholesterol, LDL-C, VLDL-C, TG, SBP. Moderately positive correlations were identified between WC, and HbA1c, HOMA-IR, testosterone, DHEAS, CRP, total cholesterol, LDL-C, VLDL-C, TG, SBP (*p* < 0.05). Weakly positive relationships were found between WC, and FBG, insulin, LH, DBP. Moreover, the correlations between ABSI, and DHEAS, total cholesterol, LDL-C, DBP were weakly positive; the correlations between ABSI, and HbA1c, CRP, VLDL-C, testosterone, TG, SBP were moderately positive (*p* < 0.05). Additionally, the following were identified: moderately positive correlations between the VAI, and DHEAS, CRP; strongly positive correlations between the VAI, and total cholesterol, LDL-C; very strongly positive correlations between the VAI, and VLDL-C, TGs (*p* < 0.05). Also, the following were found: weakly positive relationships between LAP, and insulin, HbA1c, HOMA-IR, DBP; moderately positive correlations between LAP, and testosterone, DHEAS, SBP; strongly positive correlations between LAP, and CRP, total cholesterol, LDL-C; and very strongly positive relationships between LAP, and VLDL-C, TGs (*p* < 0.05). In addition, the following were found: moderately positive correlations between the DAI, and DHEAS, CRP, DBP; strongly positive correlations between the DAI, and total cholesterol, LDL-C; and very strongly positive correlations between the DAI, and VLDL-C, TGs. Also, weakly positive relationships between the BRI, and FPG, DBP, and moderately positive relationships between the BRI, and insulin, HbA1c, HOMA-IR, testosterone, DHEAS, CRP, total cholesterol, LDL-C, VLDL-C, TGs, SBP were established (*p* < 0.05). Furthermore, moderately negative correlations between SHBG, and the BMI, WC, VAI, LAP, DAI, BRI, and strongly negative correlations between HDL-C, and the BMI, WC, VAI, LAP, DAI, BRI were determined (*p* < 0.05).

### 3.5. Assessing the Ability of Indices to Predict the Existence of PCOS and MetS

**Figure 1:**
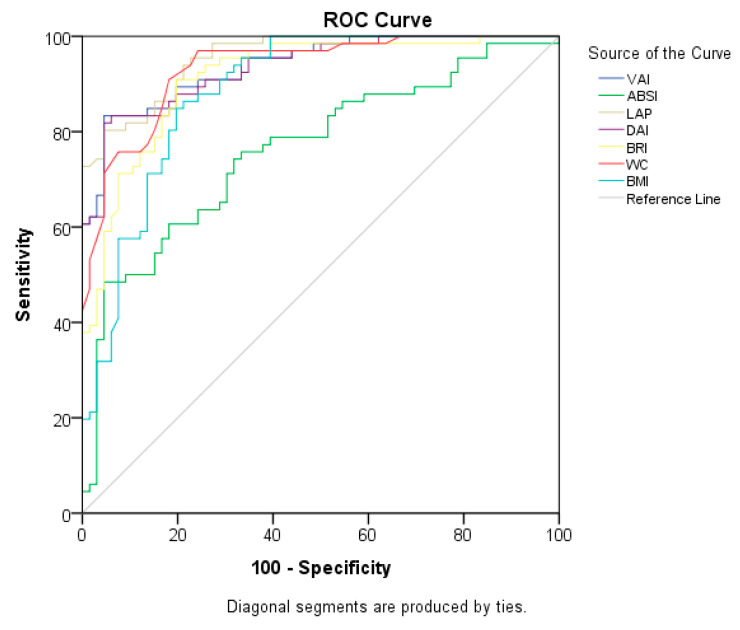
ROC curves of anthropometric measurements and indices for predicting PCOS.

The ROC curves of anthropometric measures and indices, which are signs of cardiometabolic risk for PCOS, are shown in Table 6 and Figure 1. The LAP index was found to have the highest explanatory value for PCOS (AUC = 0.956), and the cut-off value was 40.3749 (sensitivity of 84.8% and specificity of 84.8%). This is followed by the VAI (AUC = 0.938), DAI (AUC = 0.935), and WC (AUC = 0.934) (*p* = 0.000 for all). The marker with the lowest discriminatory ability for PCOS was ABSI (AUC = 0.762) (*p* = 0.000).

**Figure 2:**
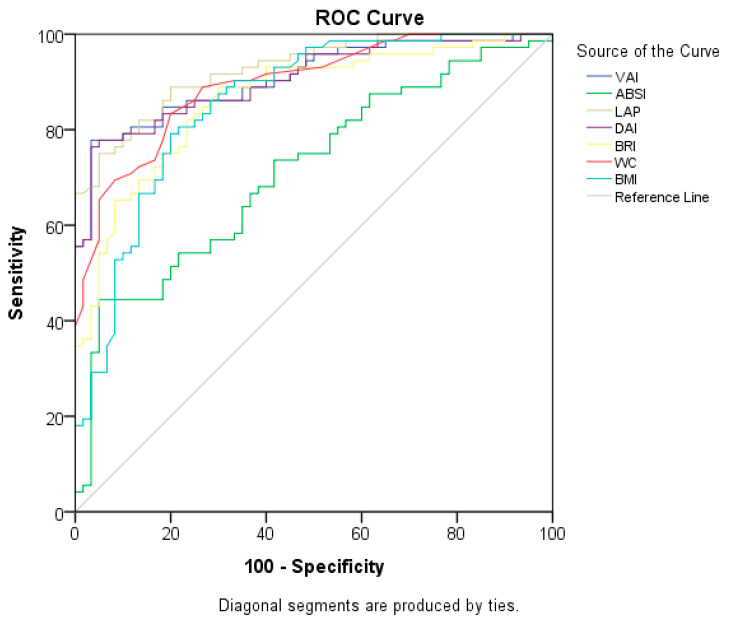
ROC curves of anthropometric measurements and indices for predicting MetS.

In Table 7 and Figure 2, the ROC curves of anthropometric measurements and indices for MetS are evaluated. The LAP index was found to have the highest discriminatory ability in terms of MetS (AUC = 0.927), and the cut-off value was 37.1488 (sensitivity of 81.9% and specificity of 81.7%) (*p* = 0.000). This is followed by the VAI (AUC = 0.908), DAI (AUC = 0.905), and WC (AUC = 0.895) (*p* = 0.000 for all). ABSI has the lowest explanatory power (AUC = 0.714; *p* = 0.000).

**Figure 3:**
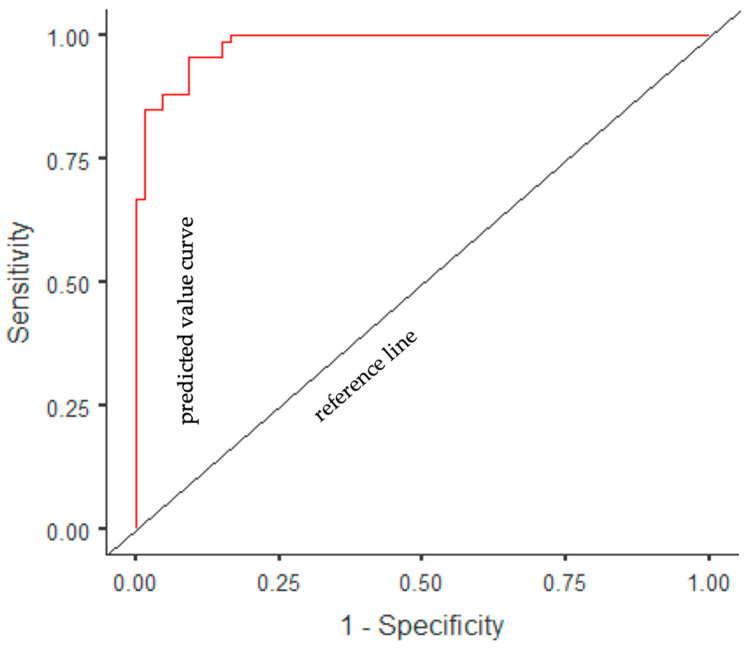
ROC curve of a multivariate logistic regression model of anthropometric measures and indices to predict PCOS.

A multivariate logistic regression model was established with the forward stepwise method, in which variables that were statistically significantly associated with the presence of PCOS were included (age, smoking, BMI, WC, ABSI, BRI, DAI, LAP index, and VAI). With the forward stepwise method, the most appropriate analysis results were obtained in the 5th step (χ2 = 43.87, *p* < 0.001), and three independent factors associated with the presence of PCOS were identified. According to the regression analysis results, the model determination coefficient was *R*^2^ (Nagelkerke) = 0.869. Accordingly, 87% of the variance in the dependent variable was explained by the independent variables. In the ROC analysis, the AUC value of the model was determined to be 0.98%; accuracy, 92%; sensitivity, 92%; and specificity, 91%. According to the multiple logistic regression model, it was determined that the probability of PCOS decreased as age increased (β = −0.15, OR = 0.86, *p* = 0.015) and the probability of PCOS increased as WC (β = 0.17, OR = 1.18, *p* < 0.001) and VAI (β = 3.17, OR = 23.83, *p* < 0.001) increased (Table 8 and Figure 3).

**Figure 4:**
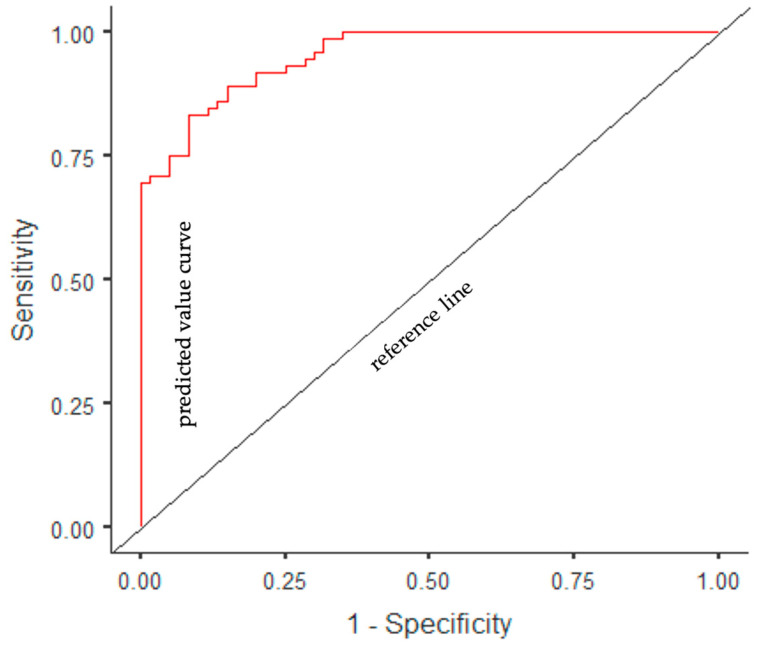
ROC curve of a multivariate logistic regression model of anthropometric measures and indices to predict MetS.

A multivariate logistic regression model was established with the forward stepwise method, in which variables that were statistically significantly associated with the presence of MetS were included (age, smoking, BMI, WC, ABSI, BRI, DAI, LAP index, and VAI). With the forward stepwise method, the most appropriate analysis results were obtained in the 3rd step (χ2 = 107.94, *p* < 0.001), and three independent factors associated with the presence of MetS were identified. According to the regression analysis results, the model determination coefficient was *R*^2^ (Nagelkerke) = 0.747. Accordingly, 75% of the variance in the dependent variable was explained by the independent variables. In the ROC analysis, the AUC value of the model was determined to be 0.95%; accuracy, 86%; sensitivity, 83%; and specificity, 88%. According to the multiple logistic regression model, it was determined that the probability of MetS decreased as the BRI increased (β = −1.88, OR = 0.15, *p* = 0.004) and the probability of MetS increased as WC (β = 0.35, OR = 1.42, *p* < 0.001) and LAP index (β = 0.09, OR = 1.09, *p* < 0.001) increased (Table 9 and Figure 4).

## 4. Discussion

Despite the high treatment costs of PCOS, which is common in women, it is reported that the number of women who cannot be diagnosed in clinical practice is high due to large differences in symptoms and clinical findings [41]. Along with ovulatory failure and hyperandrogenism, PCOS also exhibits cardiometabolic characteristics such as insulin resistance (IR) and accumulation of central body fat [7,8,43,48]. Therefore, PCOS is a systemic metabolic condition with high prevalence of metabolic disorders in addition to being a reproductive disorder [49,50].

Gender, age, ethnicity, smoking, alcohol use status, level of physical activity, and genetic predisposition all have an impact on body fat composition and visceral adipose tissue distribution [51]. Smoking, alcohol use, and a sedentary lifestyle are risk factors for PCOS and MetS [52,53], and in this study, it was observed that smoking and alcohol use habits were higher in women with PCOS (56.1% vs. 10.6%, *p* < 0.001). According to reports, 53.9% of women in Turkey do not get enough physical exercise, which is defined as 150 min of moderate-intensity activity each week [54], and the frequency of regular physical activity was found to be low in both groups in this study (22.7% vs. 22.7%, *p* = 1.000).

Obesity is reported in 30–75% of women diagnosed with PCOS [55,56], and 50–70% have varying degrees of IR [57,58]. In addition to having a higher frequency of obesity than women without PCOS, women with PCOS also have a different form of adiposity [59,60]. According to reports, women with PCOS are more likely to accumulate fat in their upper bodies than in a weight- and BMI-matched control group [61,62]. Coşar et al. (2008) and Godoy-Matos et al. (2009) stated that this effect was similar in thin women with PCOS [59,63]. Due to the release of many bioactive hormones and molecules from VAT in women or hormonal changes, PCOS itself may lead to the development of systemic inflammation [64,65,66]. Because of this reciprocal connection, there is disagreement about which condition arises first: PCOS or obesity [65,67]. As a result, increased systemic inflammation has been reported to lead to IR, hyperglycemia, hypertension, disturbed lipid profile, and non-communicable diseases such as MetS, CVD, T2DM, and non-alcoholic fatty liver disease [65,68]. Consistently with the literature, in this study, the prevalence of chronic diseases (68.2% vs. 34.8%, *p* < 0.001), including MetS (98.5% vs. 10.6%, *p* < 0.001), was found to be higher in the PCOS group than in the control group, and so was obesity (86.4% vs. 21.2%, *p* < 0.001). The cardiometabolic and adiposity features of PCOS and MetS increase the incidence [41]. According to the meta-analysis and meta-regression of the results of epidemiological studies on MetS such as METSAR [69], CREDIT [70], Chronic Disease and Risk Factors Study in Turkey [71], and Gündoğan et al.’s study [72] (which evaluated cardiovascular risk factors in Turkey (*n* = 34,893)), the prevalence rates of MetS were reported as 38.3% in women, 26.8% in men, and 32.9% in the whole population, according to ATP III criteria [73]. The prevalence of MetS is higher in patients with PCOS, and although different rates of MetS have been reported in women with PCOS in different studies, the risk of MetS increases 14 times in obese PCOS patients [74,75]. The reason why the frequency of MetS was found to be higher in patients with PCOS included in this study than in other studies may be because the participants were selected among the patients who were referred to the nutrition and diet clinic for nutritional counseling.

According to the Rotterdam criteria, the prevalence of PCOS was 19.9% in Turkey, although the frequency of MetS was reported to be 10.3% [76]. In this study, in line with the prevalence of obesity, even after adjusting for age, smoking, and alcohol consumption, except for FPG and HDL-C, all other biochemical parameters in the MetS diagnostic criteria, SBP, and DBP were found to be higher in the PCOS group than in the control group. Another common metabolic abnormality is dyslipidemia in individuals with PCOS. In the study by Başar Gökçen et al. (2021), the HDL-C levels of women with PCOS were lower than those of healthy women (*p* < 0.05) [3]. In our study, PCOS patients had higher total cholesterol, LDL-C, VLDL-C, and TGs, and lower HDL-C levels after adjusting for age, smoking, and alcohol consumption (*p* < 0.001). This difference in the frequency of MetS among studies is thought to be because all women with PCOS in this study were overweight or obese. A study has reported that a one-unit change in the BMI is associated with a change in HDL-C of 0.69 mg/dL in young adult women [77], and according to the TEKHARF study, approximately 7.5% of deaths in adults in Turkey are due to metabolic diseases associated with abdominal obesity [78].

In patients with PCOS, obesity and hyperinsulinemia are accompanied by a decrease in SHBG levels, and testosterone, predominantly in the free form unbound to SHBG, increases [79]. Accordingly, studies have reported that the increase in free testosterone levels may be 84% and an increase in DHEAS levels may be 38% in patients with PCOS and hyperandrogenemia [80,81,82]. In this study, testosterone and DHEAS levels were greater in the PCOS group, whereas SHBG levels were lower (*p* < 0.05). In addition, an increase in the ratio of LH and LH:FSH has been reported in women with PCOS [43]. In this study, although it was determined that LH and FSH levels were higher in women with PCOS, there was no statistical significance after adjusting for age, smoking, and alcohol consumption.

According to evidence-based guidelines and the Androgen Excess Society, early detection and management of PCOS and MetS reduce the incidence and severity of cardiometabolic complications in the long term [7,8,83]. For this purpose, different studies have been conducted on the role of anthropometric measurement methods in determining risk. Although CT and MRI are the most precise methods for detecting visceral adiposity, these methods require expensive, sophisticated tools and are unsuitable for application in clinical settings [65]. At this point, predicting PCOS and MetS with simple and cost-effective anthropometric indices may allow patients to be diagnosed in an early stage, start their treatment, improve their health, and increase their quality of life [8,84]. The two most common anthropometric measures are the BMI and WC. The BMI cannot distinguish between fat and muscle mass and cannot determine fat distribution [84]. However, in Ortega et al.’s (2016) study evaluating the BMI and total body fat in terms of CVD mortality, it was reported that the BMI could be as accurate as or even more important than total adiposity measurements evaluated using simple and inexpensive, and complex and expensive methods [85]. Due to the limitations of the BMI, WC measurement is recommended for the determination of central obesity. There is no consensus on a single measure for the determination of adiposity and the health risks associated with adiposity [86]. When the BMI is >35 kg/m^2^, the WHO suggests focusing on WC to predict cardiometabolic illness [87]. In this study, four out of five women were obese, and the mean BMI (32.59 ± 3.09 kg/m^2^ vs. 26.95 ± 3.42 kg/m^2^, *p* < 0.001) and WC (103.71 ± 10.33 cm vs. 84.78 ± 7.55 cm, *p* = *p* < 0.001) of the PCOS group were higher than those of the control group. Behboudi-Gandevani et al. (2016) and Tehrani et al. (2014) found similar results in their study [41,84]. Also, it was determined that the BMI and WC were correlated with biochemical parameters, consistently with the diagnosis of PCOS and MetS. In addition, the BMI and WC were found to be statistically significant indicators in the detection of PCOS and MetS in this study. While BMI > 30.41 kg/m^2^ and WC > 91.5 cm indicated the risk of PCOS (AUC = 0.887 and AUC = 91.50 cm, respectively; *p* = 0.000), BMI > 30.18 kg/m^2^ and WC > 90.5 cm increased the risk of MetS (AUC = 0.857 and AUC = 0.895, respectively; *p* = 0.000). In addition, according to the established multivariate logistic regression model, WC was found to be one of the strongest independent predictors of both PCOS and MetS.

Body fat mass, lean body mass, body fat percentage, and all anthropometric indices assessed with BIA were found to be greater in the PCOS group, as were the BMI and WC (*p* < 0.05). Contrary to our study, Kaluzna et al. (2021) reported that there were no differences in the BMI, WC, and total body fat between the PCOS group and healthy group, in which body composition and fat distribution were evaluated with DXA [65]. This difference is thought to be because the mean BMI values of both the PCOS and control groups (23.88 ± 8.02 kg/m^2^ vs. 23.05 ± 6.58 kg/m^2^, *p* > 0.05) were within the normal range. Like our study, Carmina et al. (2007) found that normal-weight and overweight women with PCOS had higher central abdominal fat (g) and central abdominal fat vs. total fat (%) DXA measurements compared with the BMI-matched control group [88]. Studies comparing the lean body mass and body fat percentage of patients with PCOS and healthy women have produced disparate results [89,90]. These discrepancies among studies’ findings have reportedly been attributed to factors such as increased body fat percentage with higher weight and WC, decreases in postprandial thermogenesis and basal metabolic rate, dysfunction of gastrointestinal hormone and appetite regulation, IR, hyperandrogenism, and physical inactivity in PCOS patients [3]. The nutritional habits of women were not evaluated in this study.

The LAP index is a marker of central fat accumulation and has been proposed as a predictor of chronic diseases [33]. Zheng and Li (2016) reported that the detection of visceral adiposity as well as general obesity may be important to assess metabolic and reproductive disorders in women with PCOS and that the VAI can be used as a complementary tool in daily practice [14]. In this study, it was determined that the LAP index and VAI had positive correlations with insulin, HOMA-IR, testosterone, DHEAS, total cholesterol, LDL-C, VLDL-C, TGs, and SBP, and negative correlations with HDL-C and SHBG (*p* < 0.05). The LAP index, VAI, and DAI were found to be more associated with lipid risk factors than the BMI, WC, BRI, and ABSI, which were related to lipid overaccumulation. Tehrani et al. (2014) reported that the LAP index and VAI were correlated with TGs and that the VAI was correlated with HDL-C in PCOS patients [41]. In another study, positive correlations were reported between the VAI, and insulin, HOMA-IR, TGs, total cholesterol, and a negative correlation with HDL-C was reported [49]. Also, in this study, the means of the LAP index and VAI were higher in the PCOS group, with the LAP index being the first (AUC = 0.956 and AUC = 0.927, respectively; *p* = 0.000 for all) and the VAI being the second highest diagnostic accuracy indices (AUC = 0.938 and AUC = 0.908, respectively; *p* = 0.000 for all) for both PCOS and MetS. The cut-off values for PCOS and MetS were detected as 40.37 and 37.14 in the LAP index, and 1.65 and 1.57 in the VAI, respectively. In addition, according to the multivariate logistic regression model, the VAI was the strongest independent predictor of PCOS, and the LAP index, for MetS. Taverna et al. (2011) and Nascimento et al. (2015) reported that the LAP index has the highest explanatory power [91,92]. According to a different study, the LAP index is a better predictor of cardiovascular risk than the BMI [14]. Ray et al. (2018) reported that the means of the BMI, WC, and LAP index were higher in the MetS group than in the control group and that the LAP index had the highest prediction accuracy [39]. Wiltgen et al. (2009) reported that the LAP index is an accepted discriminator of IR for both the PCOS group and the healthy group and that a LAP index of ≥34.5 is an additional risk factor for CVD in PCOS patients [34]. Macut et al. (2016) reported that the LAP index was higher in women with PCOS and could be used as a marker for MetS, with a lower cut-off value (25.9) than that in our study [12]. Knowles et al. (2011) also reported that the VAI was the strongest and the weight–height ratio (WHR) the lowest predictors of MetS in the normal population [40]. According to research, the LAP index and VAI are accurate and easily recognized techniques for assessing cardiovascular risk in PCOS patients [34,35,36]. Başar Gokcen et al. (2021) reported that the mean LAP index and VAI were higher in overweight/obese PCOS patients (cut-off values: 32.97 and 1.65, respectively) and that there was no difference between PCOS patients with normal BMI values and the healthy group [3]. In another study with a similar methodology, the mean VAI was found to be higher in overweight/obese women with PCOS [9]. In the study by Tehrani et al. (2014), the BMI, WC, WHR, LAP index, and VAI were compared in the detection of IR in women with and without PCOS [41]. The LAP index was reported to have the highest sensitivity; WC and the VAI were found to have the highest AUC values (0.67 and 0.66, respectively); the LAP index and VAI were reported as important predictors in cases with PCOS. The cut-off of 33.8 for the LAP index and that of 1.8 for the VAI have been suggested for the detection of IR [41], and these cut-off values are lower than those in our study. Banu et al. (2022) also reported that the LAP index for IR and the VAI for MetS were more explanatory in underweight PCOS patients [37].

Although the LAP index and VAI are examined more frequently, the number of studies in which the DAI, BRI, and ABSI are studied together and multiple indices are compared is low; as far as we know, there is no study evaluating the relationship between PCOS and the DAI. In a study evaluating the relationship between cardiometabolic diseases, and the VAI and DAI in healthy individuals, it was reported that a DAI value of ≥1.65 was associated with chronic diseases [24]. In another study in which MetS was evaluated according to IDF criteria, the DAI and LAP index were determined to be the highest (cut-offs: 0.97 and 44.52, respectively), and ABSI (cut-off: 0.075) was determined to be the index with the lowest explanatory power [3]. In this study, the cut-off values determined for the DAI and LAP index were lower for both PCOS and MetS.

To determine which anthropometric indicators are the best for determining MetS, a study of 418,343 Hispanic individuals examined 15 indices, including WC, WHR, VAI, DAI, BRI, and ABSI [31]. All anthropometric indices had AUC values greater than 0.7 except for ABSI, and while the most confident indices according to ATPIII and JIS criteria were Deurenberg fat mass in women and the VAI in men, according to IDF criteria, metabolic scores for visceral fat (METS-VF) in women and WC in men have been reported [31]. In another study conducted among 10,000 people in Iran, the predictors of MetS according to the VAI, BRI, ABSI, and IDF criteria were evaluated, and the VAI was found to be the best predictor of MetS in women (AUC = 0.866, 83.1% sensitivity, 70.0% specificity). The AUC of the BRI was 0.754, and that of ABSI was 0.491, with the lowest explanatory value [93]. It is thought that the discriminatory power is high in the studies due to the use of TGs and HDL-C parameters, which are among the MetS criteria, in the LAP index and VAI formulations. In the study by Jabczyk et al. (2023), glycemic parameters and various indices, including the LAP index, VAI, BRI, and ABSI, were evaluated in PCOS patients [94]. ABSI was reported to have the lowest significance, while the BMI and WC were significantly correlated with glycemic parameters; the LAP index and VAI were found to have a significant relationship with metabolic disorders [94]. In this study, according to the multivariate logistic regression model, the BRI was one of the strongest independent predictors of MetS.

In a study conducted in Turkey evaluating the importance of the LAP index, VAI, BRI, and ABSI to determine cardiometabolic risk and IR in PCOS patients, carotid intima–media thickness was found to have the highest explanatory power. Among the anthropometric indices, the VAI and LAP index were the highest, while ABSI and WHR were the lowest predictors [29]. Although Krauker and Krauker (2014) reported that ABSI, which they developed as an abdominal adiposity marker using WC and the BMI, is a more reliable marker for premature mortality than other anthropometric measurements [95], ABSI seems to have a low effect on explaining metabolic risk factors, including in this study [3,29,84,94].

There are very few studies evaluating multiple anthropometric indices in determining the risk of MetS in PCOS patients, and in this study, the relationship between the indices and biochemical parameters is also discussed. However, the limitations of the study include the fact that it was conducted in a single center, the sample size was small and cross-sectional, and the PCOS phenotype was not assessed. In addition, although individuals’ regular physical activity status was questioned within the scope of the study, their physical activity levels were not evaluated. Similarly, the fact that the participants’ nutritional habits were not evaluated before they applied to the nutrition clinic constitutes another limitation of the study. Studies that also evaluate individuals’ daily physical activity and dietary intake in the future may allow more detailed results to be obtained in the evaluation of PCOS and MetS. According to the results of this study, in which different anthropometric indices were evaluated together, these indices may enable the diagnosis of MetS and PCOS to be made earlier. To define the cut-off values of these indices more precisely, future studies with larger samples and evaluating the nutritional status of individuals are needed.

## 5. Conclusions

As a result, anthropometric indices can be used to determine MetS in women with PCOS, and according to the results of multivariate logistic regression models, the VAI and WC are strong predictors of PCOS, and WC, LAP index, and BRI are strong predictors of MetS. It is thought that the use of different anthropometric indices in the detection of PCOS and MetS, in addition to being simple and cost-effective, will enable the patients to have disease detected earlier, start treatment in the early period, and thus have their quality of life increased and the treatment costs reduced by preventing possible complications. The use of these anthropometric indices may provide a protective effect on public health. While evaluating the importance of different indices in determining cardiometabolic risks, there is a need for more studies conducted with larger samples and evaluating the nutritional and physical activity characteristics that have an impact on these risks, and even the change in these parameters as a result of follow-up and treatment.

## Figures and Tables

**Table 1:** Detailed information about the formulas of indices and examples from the literature results.

A body shape index (ABSI) [22]
*ABSI* = *WC* (m)/[*BMI*^2/3^ (kg/m^2^) *height*^1/2^ (m)]
Moderate correlation with WC, stronger correlation with visceral fat [22]. An effective predictor of MetS in women with PCOS [22]. Significant association with the inflammatory status in chronic kidney disease patients [27]. Underperformed in predicting chronic diseases [28].
Body roundness index (BRI) [23]
$BRI=364.2-365.5\;\times \;\sqrt{1-\left[\frac{\left(WC\;\left(\mathrm{cm}\right)/2\pi \right)\;{)}^{2}}{{\left(0.5\;\times \;Height\;\left(\mathrm{cm}\right)\right)}^{2}}\right]}$
Used to quantitatively measure individual body shape without regard to height, and a marker of VAT and body fat percentage [23]. A marker for determining the cardiometabolic risk in women with PCOS [29]. An effective predictor of type 2 diabetes [30].
Dysfunctional adiposity index (DAI) [24]
$DAI\;Female=\left(\frac{WC\;\left(\mathrm{cm}\right)}{24.02\;+\;(2.37\;*\;BMI\;\left(\mathrm{kg}/m^{2}\right)}\right)\left(\frac{TG\;\left(\mathrm{mmol/L}\right)}{1.32}\right)\left(\frac{1.43}{HDL\;-\;C\;\left(\mathrm{mmol/L}\right)}\right)$
Created as a preliminary indicator of cardiometabolic abnormalities based on adipocyte morphofunctional abnormalities [24]. A determiner of metabolic syndrome in women with PCOS [31]. Association with insulin resistance and B-cell dysfunction in people who are at high risk of developing diabetes yet appear to have no symptoms [32].
Lipid accumulation (LAP) index [25]
*LAP female* = [*WC* (cm) *−* 58] × *TG* (mmol/L)
A marker of fasting triglyceride level and intra-abdominal fat reserves [25]. A simple predictor of CV disease, because it estimates lipid excess accumulation in adults [14,25,33,34,35,36]. Linked to glucose tolerance and MetS in patients with PCOS, as well as a significant determinant of MetS [12]. An accepted discriminator of IR for both the PCOS group and the healthy group [34,37]. Predictive ability for identifying chronic kidney disease [38]. A strong predictor of MetS [39].
Visceral adiposity index (VAI) [26]
$VAI\;female=\left(\frac{WC\;\left(\mathrm{cm}\right)}{36.58\;+\;(1.89\;*\;BMI\;\left(\mathrm{kg}/m^{2}\right)}\right)\left(\frac{TG\;\left(\mathrm{mmol}/L\right)}{0.81}\right)\left(\frac{1.52}{HDL\;-\;C\;\left(\mathrm{mmol}/L\right)}\right)$
An accurate and easily recognized technique for assessing cardiovascular risk in PCOS patients [34,35,40] and also healthy subjects [41]. An indicator to evaluate metabolic and reproductive disorders in women with PCOS [14]. A predictive value in diagnosing non-alcoholic fatty liver disease and non-alcoholic steatohepatitis [42]. A strong predictor of MetS in the normal population [40].

ABSI: a body shape index; BMI: body mass index; BRI: body roundness index; CV: cardiovascular; DAI: dysfunctional adiposity index; IR: insulin resistance; LAP: lipid accumulation product index; MetS: metabolic syndrome; PCOS: polycystic ovary syndrome; VAI: visceral adiposity index; WC: waist circumference.

**Table 2:** Evaluation of demographic characteristics of participants.

	PCOS (+) (*n* = 66)		Control (*n* = 66)		*p*-Value
	Mean ± SD	Median (Min–Max)	Mean ± SD	Median (Min–Max)	
Age (year)	30.98 ± 7.52	31 (18.00–45.00)	36.00 ± 7.20	37.5 (19.00–48.00)	<0.001 *^a^
	*n*	%	*n*	%	
BMI					<0.001 *^b^
Normal	0	0.0	16	24.2	
Overweight	9	13.6	36	54.5	
Obese	57	86.4	14	21.2	
MetS					
MetS (−)	1	1.5	59	89.4	<0.001 *^b^
MetS (+)	65	98.5	7	10.6	
Chronic disease					
Yes	45	68.2	23	34.8	<0.001 *^b^
No	21	31.8	43	65.2	
Chronic disease type					
CVD	5	7.6	1	1.5	0.208 ^b^
Type 2 diabetes	19	28.8	2	3.0	<0.001 *^b^
Hypercholesterolemia	7	10.6	2	3.0	0.164 ^b^
Hypertriglyceridemia	2	3.0	0	0.0	0.496 ^b^
Hypertension	19	28.8	4	6.1	0.001 *^b^
Rheumatic disease	3	4.5	2	3.0	0.999 ^b^
Kidney disease	2	3.0	1	1.5	0.999 ^b^
Gastrointestinal disease	6	9.1	5	7.6	0.999 ^b^
Other	1	1.5	0	0.0	0.999 ^b^
Smoking					
Yes	21	31.8	7	10.6	0.003 *^b^
No	45	68.2	59	89.4	
Alcohol consumption					
Yes	28	42.4	0	0.0	<0.001 *^b^
No	38	57.6	66	100.0	
Smoking/alcohol					
Yes	37	56.1	7	10.6	<0.001 *^b^
No	29	43.9	59	89.4	
Regular physical activity					
Yes	15	22.7	15	22.7	1.000 ^b^
No	51	77.3	51	77.3	

CVD: cardiovascular disease; MetS: metabolic syndrome; PCOS: polycystic ovary syndrome; SD: standard deviation. * *p* < 0.05; ^a^ independent-samples *t*-test; ^b^ chi-square test.

**Table 3:** Evaluation of anthropometric measurements and indices.

	PCOS (+) (*n* = 66)		Control (*n* = 66)				
Anthropometric Measurement and Indices	Mean ± SD	Median (Min–Max)	Mean ± SD	Median(Min–Max)	t ^a^/Z ^b^	*p*-Value	Adjusted *p*-Value **
BMI (kg/m^2^)	32.59 ± 3.09	32.29 (27.12–39.78)	26.95 ± 3.42	26.43 (21.19 ± 35.01)	−9.918 ^a^	<0.001 *	<0.001 *
WC (cm)	103.71 ± 10.33	103.50 (82.00–128.00)	84.78 ± 7.55	85.00 (71.00–105.00)	8.605 ^b^	<0.001 *	<0.001 *
Body fat mass (kg)	38.90 ± 10.76	37.25 (18.40–68.60)	28.87 ± 9.30	27.80 (13.00–59.10)	5.359 ^b^	<0.001 *	<0.001 *
Lean body mass (kg)	50.69 ± 8.22	49.55 (11.50–78.90)	47.82 ± 4.26	47.10 (37.60–60.80)	3.141 ^b^	0.014 *	0.041 *
Body fat percentage (%)	41.76 ± 5.45	42.35 (26.90–51.60)	37.23 ± 6.85	37.60 (22.20–65.00)	−4.203 ^a^	<0.001 *	0.001 *
ABSI	0.0798 ± 0.0065	0.0797 (0.0603–0.0933)	0.0738 ± 0.0058	0.0742 (0.0605–0.0906)	5.518 ^a^	<0.001 *	<0.001 *
BRI	6.44 ± 1.72	6.30 (2.74–10.83)	3.73 ± 1.10	3.50 (2.06–6.70)	8.236 ^b^	<0.001 *	<0.001 *
DAI	2.11 ± 1.52	1.80 (0.51–8.53)	0.64 ± 0.27	0.56 (0.26–1.41)	8.615 ^b^	<0.001 *	<0.001 *
LAP	87.21 ± 43.97	85.51 (24.00–249.14)	24.66 ± 12.21	21.85 (8.95–57.29)	9.041 ^b^	<0.001 *	<0.001 *
VAI	3.77 ± 2.73	3.26 (0.92–15.39)	1.11 ± 0.48	0.99 (0.45–2.49)	8.693 ^b^	<0.001 *	<0.001 *

ABSI: a body shape index; BMI: body mass index; BRI: body roundness index; DAI: dysfunctional adiposity index; LAP: lipid accumulation product index; PCOS: polycystic ovary syndrome; SD: standard deviation; VAI: visceral adiposity index; WC: waist circumference. * *p* < 0.05; ** adjusted for age and smoking/alcohol; ^a^ independent-samples *t*-test; ^b^ Mann–Whitney U test.

**Table 4:** Evaluation of biochemical parameters and blood pressure measurements.

	PCOS (+) (*n* = 66)		Control (*n* = 66)				
	Mean ± SD	Median (Min–Max)	Mean ± SD	Median (Min–Max)	t ^a^/Z ^b^	*p*-Value	Adjusted *p*-Value **
FPG (mg/dL)	96.69 ± 12.49	95.00 (75.00–151.00)	91.51 ± 7.76	91.00 (74.00–112.00)	2.539 ^b^	0.011 *	0.190
Insulin (mg/dL)	11.18 ± 3.39	11.40 (4.20–19.60)	9.02 ± 3.52	7.90 (3.10–17.70)	3.624 ^b^	<0.001 *	0.004 *
HbA1 c (%)	5.53 ± 0.61	5.45 (4.20–8.50)	5.21 ± 0.39	5.20 (4.20–6.20)	3.651 ^b^	<0.001 *	0.025 *
HOMA-IR	2.71 ± 1.04	2.42 (0.94–6.75)	2.04 ± 0.84	1.77 (0.73–4.24)	3.754 ^b^	<0.001 *	0.003 *
FSH (mlU/mL)	7.15 ± 2.67	7.07 (2.23–11.49)	5.87 ± 2.38	6.10 (0.43–10.29)	2.713 ^b^	0.007 *	0.069
LH (mlU/mL)	8.40 ± 3.26	7.47 (4.50–23.42)	7.60 ± 3.63	7.07 (0.37–23.42)	1.385 ^b^	0.166	0.256
SHBG (nmol/L)	32.94 ± 16.36	33.84 (10.16–75.80)	68.22 ± 48.66	49.40 (12.20–229.00)	−5.554 ^b^	<0.001 *	<0.001 *
Testosterone (ng/mL)	85.34 ± 31.41	80.50 (36.00–174.00)	60.00 ± 10.65	60.00 (36.00–101.00)	5.056 ^b^	<0.001 *	<0.001 *
DHEAS (mcg/dL)	311.12 ± 109.00	307.00 (164.00–778.00)	247.53 ± 126.57	214.50 (111.00–778.00)	4.482 ^b^	<0.001 *	0.011 *
LH:FSH ratio	1.41 ± 0.88	1.10 (0.39–4.60)	1.53 ± 1.04	1.33 (0.05–6.56)	−0.856 ^b^	0.392	0.553
CRP (mg/L)	2.25 ± 1.38	2.05 (0.0–5.80)	0.92 ± 0.62	0.80 (0.0–4.50)	6.445 ^b^	<0.001 *	<0.001 *
Total cholesterol (mg/dL)	213.87 ± 43.15	212.50 (109.00–315.00)	182.57 ± 35.06	182.00 (106.00–263.00)	−4.573 ^a^	<0.001 *	<0.001 *
HDL-C (mg/dL)	43.45 ± 9.94	44.00 (14.00–71.00)	60.00 ± 10.65	60.00 (36.00–101.00)	9.227 ^a^	<0.001 *	<0.001 *
LDL-C (mg/dL)	137.34 ± 38.89	137.50 (41.00–239.00)	111.60 ± 31.34	109.00 (47.00–190.00)	−4.186 ^a^	<0.001 *	<0.001 *
VLDL-C (mg/dL)	33.81 ± 15.46	32.50 (10.00–85.00)	16.17 ± 6.43	14.50 (6.00–35.00)	7.403 ^b^	<0.001 *	<0.001 *
TGs (mg/dL)	169.26 ± 78.26	160.00 (52.00–424.00)	81.88 ± 31.98	76.50 (36.00–174.00)	7.314 ^b^	<0.001 *	<0.001 *
SBP (mmHg)	129.18 ± 13.14	130.00 (80.00–155.00)	116.21 ± 11.69	117.50 (98.00–140.00)	5.740 ^b^	<0.001 *	<0.001 *
DBP (mmHg)	81.46 ± 8.50	80.00 (60.00–110.00)	74.46 ± 8.04	73.50 (60.00–100.00)	4.872 ^b^	<0.001 *	<0.001 *

CRP: C-reactive protein; DBP: diastolic blood pressure; DHEAS: dehydroepiandrosterone sulfate; FPG: fasting plasma glucose; FSH: follicle-stimulating hormone; HbA1c: hemoglobin A1c; HDL-C: high-density lipoprotein cholesterol; HOMA-IR: homeostasis model assessment of insulin resistance; LH: luteinizing hormone; LDL-C: low-density lipoprotein cholesterol; PCOS: polycystic ovary syndrome; SD: standard deviation; SHBG: sex hormone-binding globulin; SBP: systolic blood pressure; TGs: triglycerides; VLDL-C: very-low-density lipoprotein cholesterol. * *p* < 0.05: ** adjusted for age and smoking/alcohol; ^a^ independent-samples *t*-test; ^b^ Mann–Whitney U test.

**Table 5:** Evaluation of the correlations among biochemical, clinical, and anthropometric measurements.

		BMI	WC	*ABSI*	VAI	LAP	DAI	BRI
FPG (mg/dL)	r	0.263	0.214	0.006	0.126	0.138	0.122	0.205
	*p*	0.002 **	0.014 *	0.941	0.150	0.115	0.165	0.018 *
Insulin (mg/dL)	r	0.299	0.249	0.075	0.212	0.245	0.206	0.272
	*p*	<0.001 **	0.004 **	0.390	0.015 *	0.005 **	0.018 *	0.002 **
HbA1c (%)	r	0.284	0.358	0.256	0.149	0.234	0.142	0.351
	*p*	0.001 **	<0.001 **	0.003 **	0.088	0.007 **	0.104	<0.001 **
HOMA-IR	r	0.338	0.268	0.060	0.216	0.251	0.210	0.287
	*p*	<0.001 **	0.002 **	0.497	0.013 *	0.004 **	0.016 *	0.001 **
FSH (mlU/mL)	r	0.069	0.131	0.065	0.153	0.158	0.153	0.127
	*p*	0.434	0.135	0.462	0.080	0.070	0.079	0.148
LH (mlU/mL)	r	0.049	0.172	0.106	0.095	0.122	0.094	0.121
	*p*	0.580	0.049 *	0.226	0.278	0.163	0.284	0.166
SHBG (nmol/L)	r	−0.305	−0.286	−0.097	−0.361	−0.328	−0.362	−0.282
	*p*	<0.001 **	0.001 **	0.268	<0.001 **	<0.001 **	<0.001 **	0.001 **
Testosterone (ng/mL)	r	0.198	0.288	0.228	0.247	0.348	0.247	0.294
	*p*	0.023 *	0.001 **	0.008 **	0.004 **	<0.001 **	0.004 **	0.001 **
DHEAS (nmol/L)	r	0.275	0.328	0.197	0.311	0.340	0.307	0.299
	*p*	0.001 **	<0.001 **	0.024 *	<0.001 **	<0.001 **	<0.001 **	0.001 **
LH:FSH ratio	r	0.032	0.035	0.007	−0.039	−0.027	−0.041	0.005
	*p*	0.717	0.692	0.937	0.657	0.762	0.642	0.955
CRP (mg/L)	r	0.391	0.461	0.290	0.491	0.511	0.488	0.440
	*p*	<0.001 **	<0.001 **	0.001 *	<0.001 **	<0.001 **	<0.001 **	<0.001 **
Total cholesterol (mg/dL)	r	0.345	0.370	0.181	0.545	0.598	0.544	0.394
	*p*	<0.001 **	<0.001 **	0.038 *	<0.001 **	<0.001 **	<0.001 **	<0.001 **
HDL-C (mg/dL)	r	−0.517	−0.538	−0.183	−0.748	−0.607	−0.745	−0.520
	*p*	<0.001 **	<0.001 **	0.036 *	<0.001 **	<0.001 **	<0.001 **	<0.001 **
LDL-C (mg/dL)	r	0.316	0.363	0.186	0.513	0.526	0.514	0.375
	*p*	<0.001 **	<0.001 **	0.033 *	<0.001 **	<0.001 **	<0.001 **	<0.001 **
VLDL-C (mg/dL)	r	0.465	0.475	0.254	0.903	0.861	0.905	0.479
	*p*	<0.001 **	<0.001 **	0.003 **	<0.001 **	<0.001 **	<0.001 **	<0.001 **
TGs (mg/dL)	r	0.485	0.485	0.256	0.943	0.903	0.945	0.497
	*p*	<0.001 **	<0.001 **	0.003 **	<0.001 **	<0.001 **	<0.001 **	<0.001 **
SBP (mmHg)	r	0.306	0.380	0.263	0.221	0.270	0.219	0.325
	*p*	<0.001 **	<0.001 **	0.002 **	0.011 *	0.002 **	0.012 *	<0.001 **
DBP (mmHg)	r	0.199	0.261	0.201	0.249	0.226	0.250	0.247
	*p*	0.022 *	0.002 **	0.021 *	0.004 **	0.009 **	0.004 **	0.004 **

ABSI: a body shape index; BMI: body mass index; BRI: body roundness index; CRP: C-reactive protein; DAI: dysfunctional adiposity index; DBP: diastolic blood pressure; DHEAS: dehydroepiandrosterone sulfate; FPG: fasting plasma glucose; FSH: follicle-stimulating hormone; HbA1c: hemoglobin A1c; HDL-C: high-density lipoprotein cholesterol; HOMA-IR: homeostasis model assessment of insulin resistance; LAP: lipid accumulation product index; LH: luteinizing hormone; LDL-C: low-density lipoprotein cholesterol; PCOS: polycystic ovary syndrome; SD: standard deviation; SHBG: sex hormone-binding globulin; SBP: systolic blood pressure; TGs: triglycerides; VAI: visceral adiposity index; VLDL-C: very-low-density lipoprotein cholesterol; WC: waist circumference. * *p* < 0.05, ** *p* < 0.01.

**Table 6:** Cut-off values of anthropometric measurements and indices for predicting PCOS.

	AUC (95%)	Cut-Off	*p*	Sensitivity (%)	Specificity (%)
BMI	0.887 (0.830–0.942)	30.41	0.000 *	80.3	80.3
WC	0.934 (0.894–0.974)	91.50	0.000 *	84.8	83.3
ABSI	0.762 (0.680–0.845)	0.0772	0.000 *	69.7	69.7
BRI	0.915 (0.868–0.963)	4.7497	0.000 *	83.3	83.3
DAI	0.935 (0.895–0.974)	0.9432	0.000 *	83.3	83.3
LAP	0.956 (0.927–0.985)	40.3749	0.000 *	84.8	84.8
VAI	0.938 (0.901–0.976)	1.6556	0.000 *	84.8	84.8

ABSI: a body shape index; AUC: area under the curve; BMI: body mass index; BRI: body roundness index; DAI: dysfunctional adiposity index; LAP: lipid accumulation product index; VAI: visceral adiposity index; WC: waist circumference. * *p* < 0.05.

**Table 7:** Cut-off values of anthropometric measurements and indices for predicting MetS.

	AUC (95%)	Cut-Off	*p*	Sensitivity (%)	Specificity (%)
BMI	0.857 (0.792–0.922)	30.18	0.000 *	79.2	80.0
WC	0.895 (0.843–0.947)	90.50	0.000 *	83.3	80.0
ABSI	0.714 (0.627–0.801)	0.0769	0.000 *	66.7	63.3
BRI	0.866 (0.804–0.927)	4.6383	0.000 *	76.4	76.7
DAI	0.905 (0.855–0.956)	0.8774	0.000 *	81.9	81.7
LAP	0.927 (0.886–0.968)	37.1488	0.000 *	81.9	81.7
VAI	0.908 (0.858–0.957)	1.5711	0.000 *	81.9	81.7

ABSI: a body shape index; AUC: area under the curve; BMI: body mass index; BRI: body roundness index, DAI: dysfunctional adiposity index; LAP: lipid accumulation product index; VAI: visceral adiposity index; WC: waist circumference. * *p* < 0.05.

**Table 8:** Multivariate logistic regression model results of anthropometric measures and indices to predict PCOS.

		95% Confidence Interval						95% Confidence Interval	
Predictor	Estimate	Lower	Upper	SE	Z	*p*-Value	Odds Ratio	Lower	Upper
Intercept	−16.415	−26.5647	−6.2661	5.1783	−3.17	0.002	7.43 × 10^−8^	2.90 × 10^−12^	0.00190
Age	−0.150	−0.2709	−0.0292	0.0617	−2.43	0.015 *	0.861	0.763	0.97119
WC	0.168	0.0700	0.2669	0.0502	3.35	< 0.001 *	1.183	1.072	1.305
VAI	3.171	1.4670	4.8749	0.8694	3.65	< 0.001 *	23.831	4.336	130.967

SE: standard error; VAI: visceral adiposity index; WC: waist circumference. * *p* < 0.01, multivariate logistic regression (forward stepwise model); *R*^2^ (Nagelkerke) = 0.869; Model *χ*2 = 43.865; *p* < 0.001).

**Table 9:** Multivariate logistic regression model results of anthropometric measures and indices to predict MetS.

		95% Confidence Interval						95% Confidence Interval	
Predictor	Estimate	Lower	Upper	SE	Z	*p*-Value	Odds Ratio	Lower	Upper
Intercept	−26.5558	−40.3005	−12.811	7.0127	−3.79	<0.001	2.93 × 10^−12^	3.15 × 10^−18^	2.73 × 10^−6^
WC	0.3468	0.1428	0.551	0.1041	3.33	<0.001 *	1.415	1.1534	1.735
BRI	−1.8832	−3.1668	−0.600	0.6549	−2.88	0.004 *	0.152	0.0421	0.549
LAP	0.0909	0.0431	0.139	0.0244	3.73	<0.001 *	1.095	1.0441	1.149

BRI: body roundness index; LAP: lipid accumulation product index; SE: standard error; WC: waist circumference. * *p* < 0.01, multivariate logistic regression (forward stepwise model); R^2^ (Nagelkerke) = 0.747; Model χ2 = 107.944; *p* < 0.001.

## Data Availability

The study did not report any data.
